# 

**Published:** 1909-02

**Authors:** 


					﻿Song of The First Lord. Lydston’s Hot Shot.
New Board of Health Bill.
A ox. XXIV.
FEBRUARY, 1909.
No. 8.
TEXAS
MEDICAL
JOURNAL
“RED BACK”
PUBLISHED AT AUSTIN, TEXAS, BY
F. E. DANIEL, M. D.,	-	- Proprietor
PRINTED BY THE VON BOECKMANN-JONES CO.
Subscription, $1.00 a Year, in Advance. -	Single Copies, 10 Cents
Entered at the Postoffice at Austin, Texas, as Second-class Mail Matter
				

